# Co‐design and user‐testing outcomes of a dementia risk reduction education program for multicultural communities in Australia, the MindCare program

**DOI:** 10.1002/alz70860_099598

**Published:** 2025-12-23

**Authors:** Josefine Antoniades, Dilnaz Bilimoria, Bianca Brijnath, Katrin Gerber, Robyn Woodward‐Kron, Pazit Levinger, Tuan Anh Nguyen, Joanne Enticott, Andrew Gilbert, Thu Ha Dang, Antonia Thodis, Lee‐Fay Low, Phuong Lan Do, Steve Vakkas, Laila Bushra, Rina Barhoum

**Affiliations:** ^1^ La Trobe University, Bundoora, VIC, Australia; ^2^ Consumer Representative, Melbourne, VIC, Australia; ^3^ RMIT, Melbourne, VIC, Australia; ^4^ The University of Melbourne, Melbourne, VIC, Australia; ^5^ National Ageing Research Institute, Parkville, VIC, Australia; ^6^ National Ageing Research Institute, Melbourne, VIC, Australia; ^7^ Monash University, Melbourne, VIC, Australia; ^8^ Swinburne University of Technology, Melbourne, VIC, Australia; ^9^ The George Institute, Melbourne, VIC, Australia; ^10^ The University of Sydney, Sydney, NSW, Australia

## Abstract

**Background:**

Current dementia prevention programs largely target Western populations, overlooking multicultural communities and exacerbating health disparities. In Australia, where over 30% of the population is overseas‐born, the *MindCare* project aims to co‐develop a culturally tailored dementia prevention program for Hindi, Vietnamese, Greek, and Arabic‐speaking communities.

**Method:**

Using co‐design methodologies, the program incorporated the nominal group technique for cultural adaptation workshops and individual consultations. The Think‐Aloud method was then applied during user testing to obtain final feedback to finalize materials.

**Result:**

The initial *MindCare* prototype, based on the *Lancet Commission: Dementia Prevention, Intervention, and Care* report, was refined through six workshops with 37 participants from target communities, experts, and service providers. Iterative adjustments improved flow, language, visuals, and transitions, with key changes addressing stigma, dietary guidance, and content sequencing.

The program was then culturally‐tailored and translated into four bilingual versions (target languages and English) through seven cultural‐adaptation workshops and 10 consultations with 50 participants. The adaptations integrated culturally specific foods, activities, and practices. Striking a balance between evidence‐based content and community preferences required negotiation in some groups, however consensus was achieved in most cases.

User testing (*n* = 3 per language, total *n* = 12) rendered minor feedback to simplify presenter notes, reduce activities, and extend break times. These insights led to 15 adjustments in the final program. Participants liked the overall layout and aesthetics of the program and found materials to be culturally sensitive, engaging, and well‐balanced in active and passive learning ratio.

**Conclusion:**

The rigorous co‐design process which involved close collaboration with nearly 100 participants from the target communities, experts, and key services ensured the programs met diverse cultural needs while maintaining evidence‐based rigor. The process of co‐designing *MindCare* was far from linear and required flexibility, and iterative feedback cycles, however, this has been key to the delivery of four culturally‐tailored community‐education programs. The final *MindCare* programs will be evaluated through a national randomized controlled trial (RCT) in 2025. Upon trial completion, *MindCare* programs will be freely available online, offering an impactful dementia prevention education resource for multicultural communities in Australia, with scope for further adaption for use in other languages or countries.

